# Evaluation of eye movements and visual performance in patients with cataract

**DOI:** 10.1038/s41598-020-66817-w

**Published:** 2020-06-18

**Authors:** Yu Wan, Jiarui Yang, Xiaotong Ren, Zitong Yu, Rong Zhang, Xuemin Li

**Affiliations:** 1 Department of Ophthalmology, Peking University Third Hospital, Beijing, China; 2 Beijing Key Laboratory of Restoration of Damaged Ocular Nerve, Peking University Third Hospital, Beijing, China; 3 Department of Neurobiology, School of Basic Medical Sciences, Peking University, Beijing, China; 4 Neuroscience Research Institute, Peking University, Beijing, China; 5 Key Laboratory for Neuroscience, Ministry of Education/National Health and Family Planning Commission, Peking University, Beijing, China

**Keywords:** Ocular motility disorders, Eye manifestations, Lens diseases

## Abstract

Eye movement is an essential component of visual perception. Eye movement disorders have been observed in many eye disease, and are thought to affect various visual performance in daily life. However, eye movement behaviors of the elderly with cataract are poorly understood, and the impact of cataract surgery on eye movements has not been investigated. In this study, we observed the eye movement behaviors in thirty patients with bilateral age-related cataract while performing three performance-based tasks (visual search, face recognition and reading). Eye movements were automatically recorded by an eye tracker during task performance. We found an overall improved visual performance postoperatively, presented as elevated percentage of correctly identified objects and faces, reduced search time and increased reading speed. Eye movement parameters were found significantly altered after cataract surgery. Fixation count, total fixation duration and total visit duration were markedly increased in the visual search task and face recognition task. The proportion of regressive saccades was obviously decreased in the reading task. These eye movement parameters were found to be correlated with the measures of visual performance. Our findings suggested a potential association between the eye movement disturbance and impaired visual performance, and provided a new insight on the potential usefulness of eye movement as an objective and valid tool to understand visual impairments caused by cataract, as well as evaluate practical outcomes of cataract surgery.

## Introduction

Cataract is the leading cause of world blindness and is one of the most common eye conditions in the aging population^1^. During the last decades, the impact of cataract on daily activities and quality of life has been widely investigated^2–4^. Previous studies have pointed out that cataract leads to difficulties in everyday activities and causes a poorer perceived quality of life^5,6^. Bilateral cataract surgery is effective to achieve notable improvements in vision-related activity and satisfaction with vision^7^. However, the impairments of everyday activities and quality of life are mostly assessed by patient-reported questionnaires, which means that the subjects themselves play a decisive role in the outcomes and the results sometimes can be susceptible to bias due to the subjective nature. Therefore, using more objective performance-based measures will be a complement to the direct assessment of a person’s visual ability in daily living.

Eye movement is a natural reflex to direct our gaze towards a visual stimulus and maintain a stable and clear image on the retina^8^. When performing everyday tasks, our eyes move to bring new information onto the fovea by performing a series of saccades, interspaced with fixations to retrieve useful information for analysis^9,10^. As an essential component of visual perception, eye movement is more objective than self-reported questionnaire when measuring the impact of eye diseases on daily activities and quality of life^11^. With this advantage, eye movement parameters have become important measures in many vision researches^12^.

Several studies have previously observed altered eye movement behaviors in visually impaired patients, and have indicated a link between the abnormal eye movements and functional difficulties in daily activities^13^. For instance, the high fixation instability in people with macular disease was suggested as the contributing factor for their poor visual acuity, face recognition and reading ability^14–19^. Similarly, increased fixation instability and altered saccadic eye movements were observed in amblyopic eyes, which were correlated with the amblyopic acuity deficit, and hence impaired performance of visual-reliant everyday tasks^20–23^. In investigations of eye movements in glaucoma, eye-tracking has become a tool to evaluate the effect of glaucomatous visual field defects on the functional ability to undertake everyday tasks^24–28^. The altered saccadic eye movements could even be found in glaucoma at very early stage without any detectable visual field deficit^29^. However, there has been little research investigating eye movements in cataract. A prior study has detected saccadic reaction time in eye movement perimetry in subjects with various grades of cataract severity, but only found altered saccadic reaction time in advanced cataract (Lens Opacities Classification System, LOCS III grade V) after the cataract surgery^30^. To our knowledge, there is no relevant study assessing the eye movement parameters in patients with cataract while performing everyday tasks, or at least surrogates of those in laboratory conditions.

In the present study, we aimed to observe the eye movement behaviors in patients with cataract during performance-based tasks including visual search, face recognition and reading tasks. Specifically, we made preoperative and postoperative comparisons of eye movement parameters, and investigated the correlation between eye movement disturbance and impaired performance in cataract.

## Methods

### Study design and participants

A prospective cohort study was conducted between June 2019 and November 2019 in Peking University Third Hospital. 30 participants were selected from the patients with bilateral age-related cataracts who planned to undergo cataract extraction by phacoemulsification and intraocular lens implantation. Patients with any cognitive deficit or other ocular comorbidity that had known effects on eye movements or visual acuity were excluded. Each patient received standard ophthalmic examinations including corrected distant visual acuity (CDVA), slit-lamp examination of lens opacity (LOCS III grading) and other necessary preoperative assessments. A separate group of 22 age-matched controls were also recruited to test the practice effect. The study conformed to the tenets of the Declaration of Helsinki, and the investigational procedures were reviewed and approved by the Institutional Review Board of Peking University Third Hospital. Informed consents were obtained from all participants. All images used in this study were approved for publication.

### Testing procedure and stimulus

The eye tracking tests were conducted in a specific ophthalmic examination room with a constant ambient luminance. Participants were required to sit 65 cm from a 21.5-inch computer monitor displaying at a resolution of 1920 × 1200 (Dell ST2220Mb; Dell Corporation, Texas, USA). The eye tracker (Tobii Pro X3-120 Eye Tracker; Tobii AB Inc., Danderyd, Sweden) was fixed on the monitor to record eye movement data during the trials. As Tobii possessed excellent accuracy and precision with a high tolerance for head movements, we did not restrict the participants’ heads with stabilizer in order to reproduce the real visual conditions in daily life and thus capture more natural eye movement behaviors. Prior to the formal testing, each participant was given a full and detailed explanation of every procedure of the test. All individuals were tested under binocular viewing conditions with the best spectacle corrected vision.

Before the testing procedures started, calibration was made using an in-built five-point procedure of the eye tracking software. A fixation target was displayed at the center of the screen for 3 s between the presentations of consecutive stimuli, and participants were required to fixate at the central target to ensure a stationary fixation location when every new trial started. The whole test consisted of the following three parts: (1) visual search, (2) face recognition, and (3) reading task.

#### Visual search

**Figure 1:**
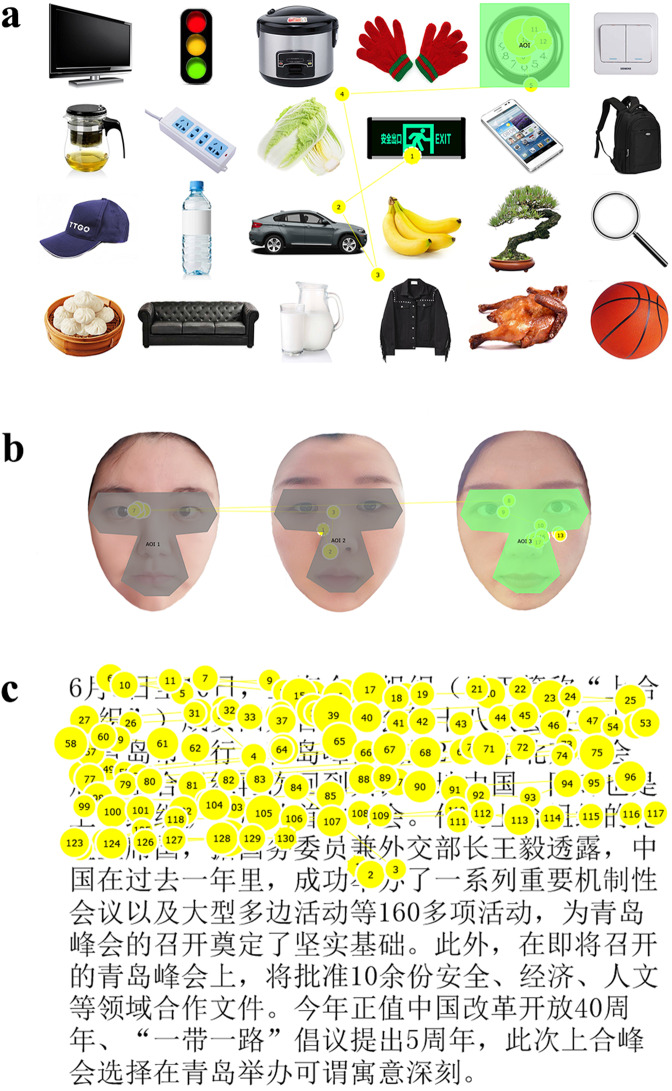
Examples of eye tracking tasks. (**a**) Example of visual search task. The gaze positions of fixations (yellow circles) and scanpaths of saccades (yellow lines) made by a participant as he were asked to find a “clock” were marked out in the figure. The size of the circles corresponded to fixation duration. The number in the circles represented the rank of fixation. Area of interest (AOI) was marked in green color. (**b**) Example of face recognition task. This is an example using the photos of researchers, for illustration purpose only. Fixations and saccades were mapped in yellow color. The target AOI was illustrated in green color, which contained the key facial features including eyes, nose and mouth. Grey color showed the invalid AOI. (**c**) Example of reading task. This example demonstrated the first passage in font size 28. The participant read sentences in a horizontal direction from left to right. The fixations were shown in circles with number, and the saccades were represented by lines.

In the visual search task, each participant was firstly given 12 s to become familiarized with 24 commonly seen objects displayed on the computer screen. Then the participant was instructed to search for a certain target item in each trial (Fig. 1a). Search time for each target was 6 s and then the scene disappeared. If the participant found the target object within the search time, he/she was required to keep fixating on it. The visual search trial was repeated 12 times, and each participant needed to search 12 items in total.

#### Face recognition

Stimuli in this part were 18 faces obtained from 9 men and 9 women of similar age. Each individual was photographed with neutral facial expression in the same lighting conditions. The face recognition task included two stages in each trial. In the viewing stage, participants were presented with a target face for 5 s and asked to memorize it. In the recognition stage, a test consisting of the previously seen face along with two other unfamiliar faces was presented (Fig. 1b). Participants were required to pick out the target face they saw previously within 5 s and kept fixating on it until it disappeared. The face recognition trial was repeated 6 times and each participant needed to recognize 3 male target faces and 3 female target faces in total.

#### Reading

The reading task was composed of four different passages including news, drug instruction, weather report and essay. Each passage was composed of 246–263 characters with 12–13 lines. The four passages were created for each font size of 28, 24, 20 and 16, respectively. All text was presented in font Song as black characters on a white background (Fig. 1c). The participants were instructed to read the passages as they normally would. Display time for each passage was 40 s. If the participant finished the whole passage before the time was up, he/she just moved their eyes away from the screen so that the following eye movements would not be recorded. Reading speed was calculated by dividing the number of characters the participant actually read by the time he/she spend reading these words. The reading trial was repeated 4 times in total. After the test, each participant was asked to talk about the general ideas of the passages to exclude invalid readings.

The whole eye tracking tests were administered in each participant twice, as the baseline assessment and the follow-up assessment. The baseline eye tracking was conducted at the final preoperative visit (for outpatients) or on the first day in hospitalization (for inpatients) (an average of 2.56 days before surgery of the first eye). After a mean recovery period of 3.43 days after the second surgery, the follow-up assessment was administered. The mean interval of the two assessments is 9.63 days. To minimize the influence of learning effect, the testing procedure remained the same but all stimuli were replaced by new items, faces and passages in the follow-up assessment.

To further determine whether the changes in visual performance and eye movements were a result of repeated testing, we also conducted the same eye tracking testing procedures in a group of age-matched healthy controls. These subjects underwent the baseline assessment and the follow-up assessment separated by a mean interval of 9.21 days, but none of them was given any intervention during this time.

### Eye tracking data

Eye movements were tracked with Tobii Pro X3-120 Eye Tracker with an accuracy of 0.4° and a sampling rate of 120 Hz. The eye tracking data was automatically recorded in the Tobii Studio eye tracking software (Tobii AB, www.tobii.com) during testing. Fixations were defined by the default fixation algorithm for Tobii Studio as stable gazes with positions remaining for at least 100 ms. Saccades were defined as the eye movements that occur between fixations^31^. To evaluate the gaze behaviors, areas of interest (AOIs) were marked out in each image using the data tools available in the Tobii Studio program. AOIs were defined using a square shape around the target object or an irregular shape surrounding the key facial features (eyes, nose and mouth) of the target face (Fig. 1). As we instructed the participant to keep fixating on the target object after he/she found it in the visual search and face recognition tasks, each successful identification was detected by analyzing the time series of his/her eye movement as follows. If a fixation located on the AOI of the target, followed by a series of gazes within the AOI, it was regarded as a successful identification. A failed identification did not have successive fixations remaining within the AOI. The percentage of correctly identified objects/faces was calculated by dividing the number of successful identifications by the number of trails.

Eye movement parameters included time to first fixation, mean fixation duration, fixation count, total fixation duration, total visit duration, progressive (forward) saccade count per line, regressive (backward) saccade count per line and percentage of regressive saccades. *Time to First Fixation* calculates how long it takes before the participant fixates on an AOI for the first time. As we instructed the participants to keep fixating on the target object/face after he/she found it, this metric was used to indicate the search time in the condition that all subsequent gazes located within the AOI. *Mean Fixation Duration* (also called *Fixation Length* in previous versions of Tobii Studio) measures the mean duration of the fixations within an AOI. *Fixation Count* calculates the number of times the participant fixates on an AOI. *Total Fixation Duration* measures the sum of the duration for all fixations within an AOI. *Total Visit Duration* (also called *Observation Length* in previous versions of Tobii Studio) measures the duration of all visits within an AOI, including fixations as well as saccades. If at the end of the recording the participant has not fixated on the AOI, the above metrics will not be computed and thus that recording will not be included in the statistics calculations. According to previous studies^13,32^, there are at least four types of reading-specific saccadic eye movements: progressive saccades (saccades that occur in a forward direction), regressive saccades (saccades that “backtrack” over previously read text), line change saccades (oblique saccades made to start a new line) and unknown saccades (other saccades that do not conform to expected reading patterns). As the meaning of the other two types of saccades during reading has not been well characterized, we specially analyzed the progressive saccades and regressive saccades in the reading section in this study.

### Statistical analysis

Statistical analyses were performed using SPSS version 23 (IBM Corp., Armonk, NY, United States) and GraphPad Prism version 8 (GraphPad Software Inc., San Diego, CA, USA). Comparisons of pre- and postoperative measures were done using paired Student’s *t*-tests (when the variables were normally distributed) or by the Wilcoxon signed-rank test (when the variables were not normally distributed). Comparisons between the cataract group and the control group were made by unpaired Student’s *t*-tests (when the variables were normally distributed) or by the Mann-Whitney U test (when the variables were not normally distributed). Spearman’s correlation coefficients were used to evaluate associations between eye movement parameters and performance defects. The Spearman correlation coefficient of less than 0.2 were considered no relationship; values of 0.2 to 0.4 suggested weak; those from 0.4 to 0.6 indicated moderate; and values of 0.6 to 0.8 were strong. Multiple linear regression analyses with stepwise selection (*P* < 0.05 as the selection criterion) were used to determine which eye movement parameters had significant influence on visual impairments.

## Results

### Demographics

This study included a cohort of 30 subjects with bilateral age-related cataract that undergone cataract surgery. There were 9 males and 21 females, who had a mean age of 70.33 ± 9.24 years (range 56–87 years). The cataract patients had an average reported cataract history of 6.3 years (range 2–25 years). The visual function characteristics of the participants were presented in Table 1. Visual acuity of both eyes significantly improved from the baseline (*P* < 0.001). Details of individual participant characteristics were presented in Supplementary Table S1.

**Table 1:** Clinical measures of visual function and visual performance in each task.

Variables	Preoperative		Postoperative		Significance
	mean ± SD	range	mean ± SD	range	
***Visual acuity***					
OD (logMAR)	0.41 ± 0.24	0.10–1.00	0.15 ± 0.14	0.00–0.50	*Z* = −4.15, *P* < 0.001
OS (logMAR)	0.48 ± 0.28	0.20–1.20	0.20 ± 0.21	0.00–0.70	*Z* = −4.57, *P* < 0.001
***LOCS III grading***					
NO, OD	3.17 ± 0.87	2.0–5.0	/	/	/
C, OD	2.43 ± 0.68	1.0–4.0	/	/	/
P, OD	1.67 ± 0.55	0.1–4.0	/	/	/
NO, OS	3.07 ± 0.83	2.0–5.0	/	/	/
C, OS	2.27 ± 0.91	1.0–4.0	/	/	/
P, OS	1.60 ± 1.16	0.1–4.0	/	/	/
***Visual search task***					
Percentage of correctly identified items (%)	75.86 ± 16.42	33.33–100.00	85.19 ± 9.90	58.33–100.00	*Z* = −1.97, *P* = 0.049
Average search time (s)	1.80 ± 0.53	0.78–2.82	1.27 ± 0.49	0.54–2.26	*t* = 4.03, *P* < 0.001
***Face recognition task***					
Percentage of correctly identified faces (%)	83.33 ± 23.13	16.67–100.00	95.24 ± 11.00	66.67–100.00	*Z* = −2.85, *P* = 0.004
Average search time (s)	1.29 ± 0.71	0.30–3.12	1.05 ± 0.65	0.44–1.05	*Z* = −1.98, *P* = 0.048
***Reading task***					
Reading speed (chars/s)	4.36 ± 1.65	1.80–7.06	5.82 ± 1.91	2.48–9.00	*t* = 6.12, *P* < 0.001

SD: standard deviation; LOCS III, Lens Opacities Classification System III; NO, nuclear opalescence; C, cortical; P, posterior subcapsular.

### Performance and eye movement parameters in visual search task

Percentage of correctly identified objects and average search time were used to evaluate the visual search performance (Table 1). The percentage of correctly identified objects before and after surgery was 75.86% and 85.19% respectively, the difference was statistically significant (*P* = 0.049). The average search time had a significant reduction from 1.80 s to 1.27 s (*P* < 0.001), indicating a quicker exploration after surgery. These results indicated an improved visual search performance of the patients after surgery.

**Figure 2:**
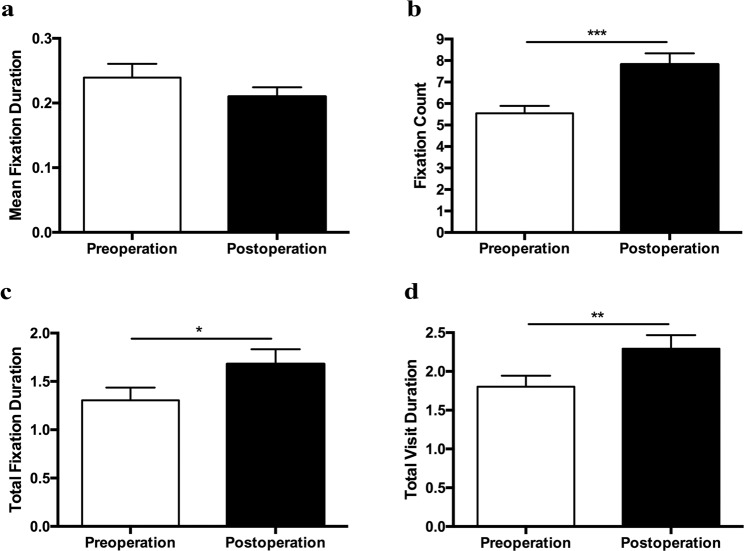
Bar graphs depicting group means of eye movement parameters in visual search task. (**a**) Mean Fixation Duration. Wilcoxon signed-rank test, *Z* = −2.00, *P* = 0.057. (**b**) Fixation Count. Paired Student’s *t*-test, *t* = 4.54, *P* < 0.001. (**c**) Total Fixation Duration. Paired Student’s *t*-test, *t* = 2.18, *P* = 0.039. (**d**) Total Visit Duration. Paired Student’s *t*-test, *t* = 2.88, *P* = 0.008. Error bars represent ± standard error of the mean (SEM). *P < 0.05; ** *P* < 0.01; *** *P* < 0.001.

Eye movement parameters were presented in Fig. 2. There were significant increases in fixation count (*P* < 0.001), total fixation duration (*P* = 0.039) and total visit duration (*P* = 0.008) after surgery, suggesting more attention on the certain target postoperatively in the visual search task. No statistically significant change was found in mean fixation duration before and after surgery.

To further investigate the relationship between the visual search performance and eye movement parameters, Spearman correlation analysis was conducted (Table 2). The percentage of correctly identified objects was weakly related to mean fixation duration (*r* = 0.291, *P* = 0.030), moderately related to fixation count (*r* = 0.558, *P* < 0.001) and strongly associated to total fixation duration (*r* = 0.645, *P* < 0.001) and total visit duration (*r* = 0.625, *P* < 0.001). Besides, moderate correlations were found between the average search time and fixation count (*r* = −0.544, *P* < 0.001), total fixation duration (*r* = −0.471, *P* < 0.001) and total visit duration (*r* = −0.532, *P* < 0.001). Multiple regression analysis demonstrated that the significant oculomotor predictors of the visual search task performance were total visit duration and fixation count (Table 3).

**Table 2:** Spearman’s correlation coefficients *r* (95% CI) of visual performance and eye movement parameters.

Variables	Visual search task		Face recognition task		Reading task
	Percentage of correctly identified items (%)	Average search time (s)	Percentage of correctly identified faces (%)	Average search time (s)	Reading speed (chars/s)
Mean Fixation Duration (s)	0.291 (0.022; 0.520) *P* = 0.030	−0.254 (−0.487; −0.013) *P* = 0.055	0.183 (−0.093; 0.431) *P* = 0.179	−0.518 (−0.689; −0.293) *P* < 0.001	−0.071 (−0.365; 0.236) *P* = 0.644
Fixation Count (n)	0.558 (0.339; 0.720) *P* < 0.001	−0.544 (−0.708; −0.326) *P* < 0.001	0.494 (0.259; 0.675) *P* < 0.001	−0.786 (−0.870; −0.658) *P* < 0.001	0.204 (−0.112; 0.482) *P* = 0.190
Total Fixation Duration (s)	0.645 (0.449; 0.781) *P* < 0.001	−0.471 (−0.657; −0.229) *P* < 0.001	0.432 (0.183; 0.629) *P* < 0.001	−0.768 (−0.859; −0.632) *P* < 0.001	0.020 (−0.283; 0.320) *P* = 0.896
Total Visit Duration (s)	0.625 (0.427; 0.776) *P* < 0.001	−0.532 (−0.669; −0.310) *P* < 0.001	0.472 (0.231; 0.659) *P* < 0.001	−0.742 (−0.842; −0.593) *P* < 0.001	0.145 (−0.164; 0.428) *P* = 0.343
Progressive saccade number per line (n)	—	—	—	—	−0.385 (−0.623; 0.083) *P* = 0.012
Regressive saccade number per line (n)	—	—	—	—	−0.328 (−0.599; 0.011) *P* = 0.051
Proportion of regressive saccades (%)	—	—	—	—	−0.321 (−0.575; 0.009) *P* = 0.039

**Table 3:** Multiple regression analyses of visual performance and eye movement parameters.

Dependent Variable	Independent variable	*B*	*SE*	*Beta*	*t*	*P*-value
***Visual search task***						
Percentage of correctly identified items (%)	Constant	60.55	4.21		14.38	*P* < 0.001
	Total Visit Duration (s)	9.60	1.88	0.58	5.11	*P* < 0.001
Average search time (s)	Constant	2.35	0.17		13.52	*P* < 0.001
	Fixation Count (n)	−0.12	0.02	−0.56	−4.96	*P* < 0.001
***Face recognition task***						
Percentage of correctly identified faces (%)	Constant	68.52	5.21		13.14	*P* < 0.001
	Fixation Count (n)	2.76	0.63	0.51	4.39	*P* < 0.001
Average search time (s)	Constant	2.97	0.23		12.60	*P* < 0.001
	Fixation Count (n)	−0.27	0.06	−1.28	−4.32	*P* < 0.001
	Mean Fixation Duration (s)	−4.61	1.24	−0.45	−3.73	*P* < 0.001
	Total Visit Duration (s)	0.54	0.23	0.77	2.36	*P* = 0.022
***Reading task***						
Reading speed (chars/s)	Constant	9.90	1.02		9.69	*P* < 0.001
	Progressive saccade number per line (n)	−0.17	0.04	−0.53	−4.00	*P* < 0.001
	Proportion of regressive saccades (%)	−0.13	0.03	−0.50	−3.75	*P* = 0.001

### Performance and eye movement parameters in face recognition task

Similar to the results of visual search task, the percentage of correctly identified faces in face recognition task was markedly improved postoperatively, from a preoperative accuracy of 83.33% to 95.24% (*P* = 0.004) (Table 1). Besides, significant reductions were found in the average search time (*P* = 0.048), suggesting a quicker face recognition after surgery.

**Figure 3:**
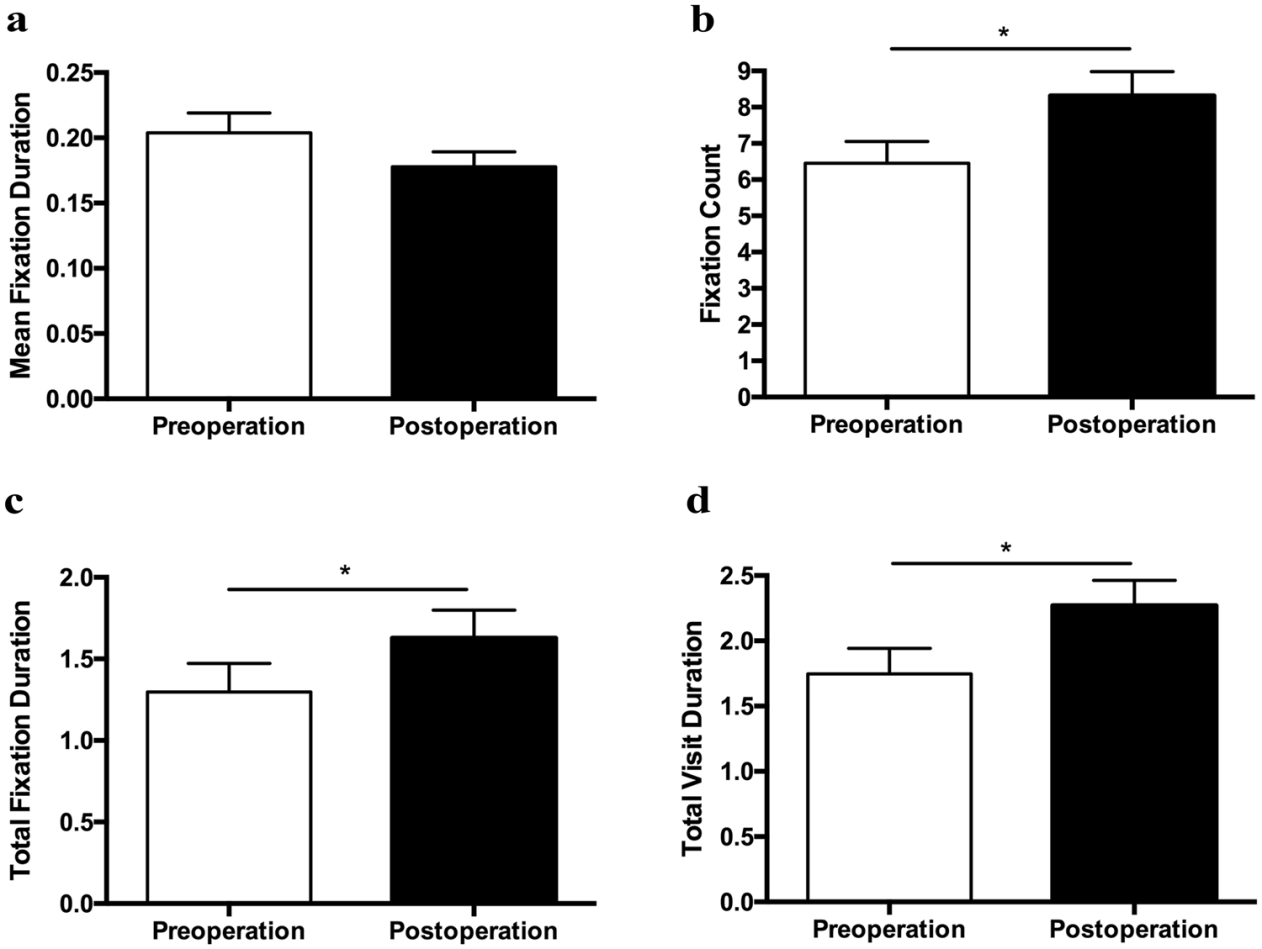
Bar graphs depicting group means of eye movement parameters in face recognition task. (**a**) Mean Fixation Duration. Wilcoxon signed-rank test, *Z* = −0.93, *P* = 0.146. (**b**) Fixation Count. Paired Student’s *t*-test, *t* = 2.49, *P* = 0.019. (**c**) Total Fixation Duration. Paired Student’s *t*-test, *t* = 2.78, *P* = 0.010. (**d**) Total Visit Duration. Paired Student’s *t*-test, *t* = 2.32, *P* = 0.028. Error bars represent ± SEM. *P < 0.05.

As illustrated in Fig. 3, number of fixations in the selected AOI was significantly increased postoperatively (*P* = 0.019). Notable increases of total fixation duration (*P* = 0.010) and total visit duration (*P* = 0.028) were also detected after surgery, indicating more attention on the target face, and thus, better face recognition performance. No significant change was found in the mean fixation duration before and after surgery.

Furthermore, correlation analysis results showed that there were moderate associations between the percentage of correctly identified faces and fixation count (*r* = 0.494, *P* < 0.001), total fixation duration (*r* = 0.432, *P* = 0.001) and total visit duration (*r* = 0.472, *P* < 0.001). The average search time was moderately associated with mean fixation duration (*r* = −0.518, *P* < 0.001), and strongly associated with fixation count (*r* = −0.786, *P* < 0.001), total fixation duration (*r* = −0.768, *P* < 0.001) and total visit duration (*r* = −0.742, *P* < 0.001). Details of the correlation analysis results were presented in Table 2. Multiple regression analysis demonstrated that the significant oculomotor predictors of the face recognition task performance included fixation count, mean fixation duration and total visit duration (Table 3).

### Performance and eye movement parameters in reading task

**Figure 4:**
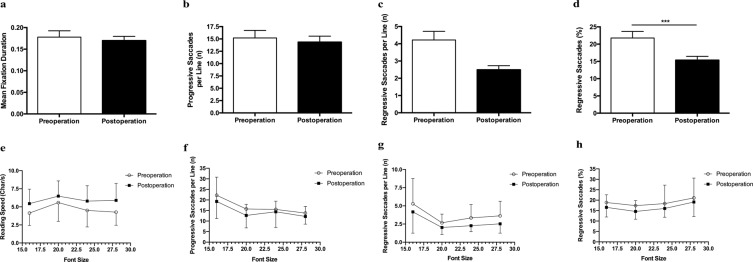
Graphs depicting group means of eye movement parameters in reading task. (**a**) Mean fixation duration. Wilcoxon signed-rank test, *Z* = −1.45, *P* = 0.354. (**b**) Number of progressive saccades per line. Wilcoxon signed-rank test, *Z* = −1.25, *P* = 0.212. (**c**) Number of regressive saccades per line. Wilcoxon signed-rank test, *Z* = −1.60, *P* = 0.110. (**d**) Proportion of regressive saccades. Wilcoxon signed-rank test, *Z* = −3.38, *P* < 0.001. Error bars represent ± SEM. Graphs of (**e**) reading speed, (**f**) number o**f** progressive saccades per line, (**g**) number of regressive saccades per line, (**h**) proportion of regressive saccades with increasing font size in baseline assessment (open circles) and follow-up assessment (filled squares). Error bars represent ± SD. ***P < 0.001.

Results of reading tasks were illustrated in Table 1 and Fig. 4. On the whole, postoperative reading speed was significantly elevated compared with preoperative baseline (*P* < 0.001), and the proportion of regressive saccades was markedly decreased (*P* < 0.001). There was no statistical difference in the number of progressive saccades or regressive saccades per line before and after surgery. No significant change of mean fixation duration was found. To further observe the reading performance in different font sizes, parameters of reading performance were illustrated with increasing font size by curves (Fig. 4). The change trend of each parameter was consistent in all font sizes before and after the operation.

Correlation analysis demonstrated that reading speed had a weak association with progressive saccade number per line (*r* = −0.385, *P* = 0.012) and proportion of regressive saccades (*r* = −0.321, *P* = 0.039) (Table 2). Multiple regression analysis showed that the progressive saccade number per line and proportion of regressive saccades were the oculomotor predictors of the reading task performance (Table 3).

### Testing for practice effect

Data of visual performance and eye movements of the control group (mean age 67.36 ± 9.15 years, 45.5% males) was shown in Supplementary Table S2. Comparisons were made between the baseline assessment and the follow-up assessment, and no significant difference was found in any parameters between the two assessments (all *P*_1_ < 0.05). This suggested that repeated testing did not have an influence on the results of the tests. The evidence of no practice effect supported that the changes in visual performance and eye movements of our cataract participants were caused by the improved visual conditions after cataract surgery.

Besides, we also compared the parameters of our cataract participants with the data from the control group. As expected, the cataract patients had worse performances than the healthy controls in the baseline assessment (*P*_2_ < 0.05). In the follow-up assessment, the postoperative average search time and total fixation duration in visual search task of the cataract participants were still worse than that of the controls, even though they had a significant improvement compared with the preoperative data. Other improved parameters of the cataract group were as good as the normal controls in the follow-up assessment (*P*_3_ < 0.05).

## Discussion

This is the first study to investigate eye movement behaviors in patients with cataract during three real-life performance-based tasks before and after cataract surgeries. We found an overall improved visual performance accompanied by alterations of several eye movements. We also detected an association between the eye movement disturbance and impaired visual performance in cataract.

Vision-related quality of life is an important issue for cataract surgery outcome evaluation^33,34^. Compared with patient-reported questionnaires, performance-based tasks measure the actual visual performance directly and are less affected by the personal subjective biases^35^. Since visual search, face recognition and reading were three of the most important everyday tasks in elder people, we evaluated the performance-based visual function in cataract patients from these three dimensions. On the basis of no practice effect, our results demonstrated that cataract surgery significantly improved the visual function and real-life visual ability of the patients. To be specific, comparing the changes from baseline to follow-up, our patients spent less time searching and correctly recognized more target items and faces in the visual search and face recognition tasks. Besides, the reading speed was significantly elevated after cataract surgery. These improvements of real-life visual performance were in accordance with previous studies, which were considered to be mainly due to the enhancement of visual acuity following cataract surgery^36,37^. Although numerous studies have investigated the changes of visual performance after cataract surgery, the underlying eye movement behaviors specific to cataract subjects remain unclear. In this study, we further examined the impact of cataract surgery on eye movement behaviors in these everyday tasks.

Our results demonstrated significant alterations of several eye movement parameters in the three types of everyday tasks after cataract surgery. Number of fixations on the target items or faces was found increased after removing the opaque lens. This was in agreement with a study which detected a 10.6% reduction in the number of fixations in the blurred condition^31^. A prior study has reported that in a face recognition memory trial, participants made fewer fixations during the tasks in which they failed to recognize a memorized face than those that led to successful recognition^38^. Similarly, we also found an improved accuracy in face recognition and visual search accompanied with the increase of fixation count, indicating enhanced eye scanning activities. Besides, total fixation durations and total visit durations were extended after surgery in our study. The longer fixation time and visit time may suggest increased awareness and visual attention on the target. It has been reported that the more fixations as well as longer fixation duration an inspector made in a visual inspection task, the more accurate his/her performance was^39^. In another study, it was reported that the total fixation duration was reduced by 10.8% with blur as compared to the best-corrected vision condition^31^. Thus, the visual condition might have a notable impact on the gaze behaviors in performance of daily activities.

Reading is a common vision-reliant ability which requires a series of saccades and fixations to move through text lines and extract information^40^. It is well documented that reading deficits of visually impaired subjects have an association with distinct differences in eye movement behaviors^13,23,41^. In this study, we found no significant change of fixation number or fixation duration in reading after surgery, which was inconsistent with previous reports that illustrated more fixations and longer fixation durations in the non-skilled readers or visually impaired readers^9,42^. Also, no correlation was detected between the reading speed and the fixation number or fixation duration in our subjects. This might be explained by a hypothesis that the fixation duration and fixation number were not strong determinants of reading speed in individuals with cataracts relative to those with other ocular disorders. Previous studies of other ocular diseases exhibited a relationship between the decreased reading rate and the increased number of saccades^23,43,44^. The additional or compensatory saccades were thought to be a vital cause of the impaired reading speed^45^. It has been reported that the glaucomatous individuals spent longer time reading a passage with their worse eye compared to the better eye, and the slower reading speed was associated with an increased proportion of regressive saccades when reading with the worse eye^32^. When a reader backtracks over previously read text more frequently, he/she makes more regressive saccades during reading, which indicates he/she has a slower reading speed and poor reading performance. In our research, we detected a notable reduction in the percentage of regressive saccades postoperatively, and found significant correlation between reading speed and proportion of regressive saccades as well as progressive saccade number. We attribute the increased postoperative reading speed to the lower proportion of backward eye movements and elevated proportion of forward eye movements through the lines of text.

Our study detected significant shorter fixation durations in the cataract population compared with the normal elderly, and the shortened fixation durations did not alter with the improved visual condition after cataract surgery. Previous studies have also reported altered fixation durations in the presence of various visual impairments, but the changes reported in different studies are not in accordance with each other. Some studies documented shorter fixation durations in glaucomatous patients compared to controls^46,47^, while some other studies in patients with strabismic amblyopia detected extended fixation durations^23,48^. Moreover, some investigations of eye movements in macular degeneration reported that fixation durations on targets were longer^49^, yet others found no change^50^. The inconsistency across studies suggested that the eye movement patterns were complex and distinct in different types of visual impairment, which still needed further investigations to understand more.

Although improvement of visual acuity is still the most widely applied outcome measurement of cataract surgery in routine clinical practice, restoration of visual ability to successfully perform everyday tasks is the ultimate goal for every patient. The usefulness of performance-based visual functional assessment is undeniable. Analyzing eye movements can help us gain more insights into the scanning behaviors during daily activities in the cataract population, and thus better understand the performance deficits in real-life tasks which cannot be completely explained by visual function alone.

### Limitations

There are several limitations in our study. First, all of our subjects were collected from the same hospital, which was located in a university area and the surrounding residents were relatively well-educated. So, it might not be able to effectively extend our findings to all individuals. Second, due to the limitation of the eye tracking device, eye movements were only examined in computer displayed two-dimensional scenes in our study, which might not exactly reflect the actual performance in the real world. Finally, we did not discuss the influence of different types of implanted intraocular lens in this study. A total of five different brands of mono-focal and multi-focal intraocular lens were applied in our patients, but no significant difference was detected in any of the eye movements parameters between the subgroups. However, this cannot rule out the possibility that the impact of different intraocular lens on eye movement behaviors did not appear due to the small sample size of each subgroups in our current study. The difference between diverse intraocular lens will be deeply investigated in our further studies.

## Conclusion

In conclusion, our study observed significantly altered eye movement behaviors accompanied with the overall improved visual performance in everyday tasks after cataract surgery. The performance-based visual function was correlated with eye movement behaviors in cataract. Our findings provide a new insight on the potential usefulness of eye movement as an objective and valid tool to understand visual impairments caused by cataract, as well as a complementary and meaningful measure to evaluate practical outcomes of cataract surgery.

## Supplementary information

Supplementary Table S1.

Supplementary Table S2.
