# Supplementary material for: Evaluation of eye movements and visual performance in patients with cataract
Source: Sci Rep. 2020 Jun 18;10:9875. doi: 10.1038/s41598-020-66817-w (PMC7303140; doi:10.1038/s41598-020-66817-w)
Supplement: Supplementary file 1 — Supplementary Table S1. [file 41598_2020_66817_MOESM1_ESM.pdf]

Evaluation of eye movements and visual performance in patients with cataract

Yu Wan[1,2#], Jiarui Yang[1,2#], Xiaotong Ren[1,2], Zitong Yu[1,2], Rong Zhang[3,4,5*], Xuemin Li[1,2*]

[1] Department of Ophthalmology, Peking University Third Hospital, Beijing, China

[2] Beijing Key Laboratory of Restoration of Damaged Ocular Nerve, Peking University Third Hospital, Beijing, China

[3] Department of Neurobiology, School of Basic Medical Sciences, Peking University, Beijing, China

[4] Neuroscience Research Institute, Peking University, Beijing, China

[5] Key Laboratory for Neuroscience, Ministry of Education/National Health and Family Planning Commission, Peking University, Beijing, China

# These authors contributed equally to this paper.

*** Correspondence:**

Xuemin Li, Department of Ophthalmology, Peking University Third Hospital, 49 North Garden Road, Haidian District, Beijing 100191, China. E-mail: lxmlxm66@sina.com. Tel: +86 13911254862. Fax Number: 8601082089951.

Rong Zhang, Department of Neurobiology, School of Basic Medical Sciences, Peking University, Beijing, China. E-mail: zhangrong@bjmu.edu.cn. Tel: 82801152.

Supplementary Table S1. Individual participant characteristics for the involved patients with cataract

No.	Age	Sex	IOL	Preoperative VA (logMAR)		Postoperative VA (logMAR)	
				OD	OS	OD	OS
1	57	F	NIDEK SZ-1	0.9	0.8	0.1	0.0
2	65	F	NIDEK SZ-1	0.3	1.2	0.5	0.6
3	70	F	Tecnis ZCB00	0.5	0.4	0.1	0.1
4	68	M	Tecnis ZCB00	0.4	0.2	0.1	0.2
5	64	F	Tecnis ZCB00	0.4	0.2	0.0	0.0
6	63	F	ZEISS AT LISA tri839MP	0.3	0.2	0.1	0.0
7	65	F	ROHTO RAY-61PL	0.3	0.2	0.1	0.2
8	78	F	Tecnis ZCB00	0.2	0.3	0.0	0.1
9	79	M	NIDEK SZ-1	0.9	0.3	0.2	0.0
10	78	F	ROHTO RAY-61PL	0.3	0.5	0.1	0.1
11	65	F	ROHTO RAY-61PL	0.4	0.5	0.1	0.1
12	84	F	Tecnis ZCB00	1.0	0.8	0.4	0.4
13	62	F	NIDEK SZ-1	0.3	0.3	0.2	0.2
14	67	F	Tecnis ZCB00	0.3	0.5	0.2	0.1
15	74	F	Tecnis ZCB00	0.5	0.3	0.1	0.2
16	77	F	ZEISS AT LISA tri839MP	0.3	0.6	0.0	0.3
17	86	M	Tecnis ZCB00	0.5	1.1	0.3	0.7
18	73	M	Tecnis ZCB00	0.2	0.2	0.0	0.1
19	64	M	NIDEK SZ-1	0.1	0.2	0.1	0.1
20	84	F	NIDEK SZ-1	0.4	0.6	0.0	0.4
21	87	M	NIDEK SZ-1	0.1	0.7	0.3	0.7
22	80	F	Lenstec SBL-3	0.4	0.4	0.4	0.3
23	61	M	ZEISS AT LISA tri839MP	0.3	0.6	0.1	0.2
24	68	F	ZEISS AT LISA tri839MP	0.1	0.3	0.0	0.0
25	56	F	ZEISS AT LISA tri839MP	0.4	0.4	0.0	0.0
26	57	F	NIDEK SZ-1	0.6	0.3	0.1	0.1
27	70	F	NIDEK SZ-1	0.5	0.7	0.3	0.0
28	76	F	ZEISS AT LISA tri839MP	0.3	0.2	0.1	0.1
29	76	M	ZEISS AT LISA tri839MP	0.9	0.9	0.1	0.6
30	56	M	Lenstec SBL-3	0.3	0.4	0.3	0.1

IOL, intraocular lens; VA, visual acuity; M, male; F, female.
