# Supplementary material for: Evaluation of eye movements and visual performance in patients with cataract
Source: Sci Rep. 2020 Jun 18;10:9875. doi: 10.1038/s41598-020-66817-w (PMC7303140; doi:10.1038/s41598-020-66817-w)
Supplement: Supplementary file 2 — Supplementary Table S2. [file 41598_2020_66817_MOESM2_ESM.pdf]

Evaluation of eye movements and visual performance in patients with cataract

Yu Wan[1,2#], Jiarui Yang[1,2#], Xiaotong Ren[1,2], Zitong Yu[1,2], Rong Zhang[3,4,5*], Xuemin Li[1,2*]

[1] Department of Ophthalmology, Peking University Third Hospital, Beijing, China

[2] Beijing Key Laboratory of Restoration of Damaged Ocular Nerve, Peking University Third Hospital, Beijing, China

[3] Department of Neurobiology, School of Basic Medical Sciences, Peking University, Beijing, China

[4] Neuroscience Research Institute, Peking University, Beijing, China

[5] Key Laboratory for Neuroscience, Ministry of Education/National Health and Family Planning Commission, Peking University, Beijing, China

# These authors contributed equally to this paper.

*** Correspondence:**

Xuemin Li, Department of Ophthalmology, Peking University Third Hospital, 49 North Garden Road, Haidian District, Beijing 100191, China. E-mail: lxmlxm66@sina.com. Tel: +86 13911254862. Fax Number: 8601082089951.

Rong Zhang, Department of Neurobiology, School of Basic Medical Sciences, Peking University, Beijing, China. E-mail: zhangrong@bjmu.edu.cn. Tel: 82801152.

Supplementary Table S2. Visual performance and eye movement parameters of the age-matched controls

Variables	Baseline (mean ± SD)	Follow-up (mean ± SD)	P_1	P_2	P_3
Visual search task					
Percentage of correctly identified items (%)	83.33 ± 18.54	85.71 ± 15.16	0.705	0.127	0.997
Average search time (s)	1.15 ± 0.56	0.95 ± 0.44	0.131	< 0.001	0.020
Mean Fixation Duration (s)	0.33 ± 0.18	0.32 ± 0.20	0.396	0.011	0.009
Fixation Count (n)	6.80 ± 2.07	7.28 ± 3.07	0.264	0.033	0.519
Total Fixation Duration (s)	1.98 ± 0.83	2.21 ± 0.19	0.145	0.004	0.004
Total Visit Duration (s)	2.49 ± 1.07	2.77 ± 1.06	0.140	0.009	0.099
Face recognition task					
Percentage of correctly identified faces (%)	95.24 ± 11.94	93.65 ± 17.06	0.715	0.010	0.859
Average search time (s)	1.12 ± 0.68	0.95 ± 0.79	0.349	0.477	0.436
Mean Fixation Duration (s)	0.31 ± 0.18	0.31 ± 0.16	0.498	0.036	< 0.001
Fixation Count (n)	6.58 ± 3.19	7.38 ± 2.59	0.075	0.898	0.279
Total Fixation Duration (s)	1.69 ± 0.97	1.83 ± 0.88	0.065	0.179	0.451
Total Visit Duration (s)	2.60 ± 1.34	2.59 ± 1.50	0.673	0.015	0.365
Reading task					
Reading speed (chars/s)	8.17 ± 3.08	8.57 ± 4.72	0.600	< 0.001	0.106
Mean Fixation Duration (s)	0.21 ± 0.05	0.22 ± 0.07	0.592	0.030	0.008
Progressive saccade number per line (n)	8.36 ± 3.98	9.28 ± 3.77	0.654	< 0.001	0.011
Regressive saccade number per line (n)	1.75 ± 2.83	2.01 ± 1.49	0.231	< 0.001	0.374
Proportion of regressive saccades (%)	13.26 ± 8.63	15.48 ± 7.36	0.576	0.005	0.962

SD: standard deviation.

P_1: baseline assessment of the control group *vs* follow-up assessment of the control group, by paired Student's t-tests (if the variables were normally distributed) or Wilcoxon signed-rank test (if the variables were not normally distributed).

P_2: baseline assessment of the control group *vs* baseline assessment of the cataract group, by unpaired Student's *t*-test (if the variables were normally distributed) or Mann-Whitney U test (if the variables were not normally distributed).

P_3: follow-up assessment of the control group *vs* follow-up assessment of the cataract group, by unpaired Student's *t*-test (if the variables were normally distributed) or Mann-Whitney U test (if the variables were not normally distributed).
